# Defoliation management and grass growth habits modulated the soil microbial community of turfgrass systems

**DOI:** 10.1371/journal.pone.0218967

**Published:** 2019-06-24

**Authors:** Qing Xia, Huaihai Chen, Tianyou Yang, Grady Miller, Wei Shi

**Affiliations:** 1 Department of Crop and Soil Sciences, North Carolina State University, Raleigh, NC, United States of America; 2 College of Life Science and Technology, Henan Institute of Science and Technology, Xinxiang, China; University of California Berkeley, UNITED STATES

## Abstract

Grass species selection and regular mowing are essential for maintaining aesthetic and environmentally sound turfgrass systems. However, their impacts on the soil microbial community, the driving force for soil N cycle and thus the environmental fate of N, are largely unknown. Here, the high throughput sequencing of 16S rRNA gene and internal transcribed spacer (ITS) region was used to evaluate how long-term defoliation management and grass growth habits (propagation types and photosynthetic pathways) modulated the soil microbial community. The investigation included three cool-season C3 grasses (creeping bentgrass, Kentucky bluegrass, and tall fescue) and three warm-season C4 grasses (bermudagrass, St. Augustinegrass, and zoysiagrass). Creeping bentgrass and bermudagrass were managed as putting greens with a lower mowing height; tall fescue spread in a tussock manner via tiller production whereas other grasses propagated in a creeping manner via rhizomes and/or stolons. Ordination analysis showed that both bacterial and fungal communities were primarily separated between putting green and non-putting green systems; and so were N-cycle gene relative abundances, with the putting greens being greater in N mineralization but lower in nitrification. Compared to warm-season grasses, cool-season grasses slightly and yet significantly enhanced the relative abundances of Chloroflexi, Verrucomicrobia, and Glomeromycota. Tall fescue yielded significantly greater bacterial and fungal richness than non-tussock grasses. As the main explanatory soil property, pH only contributed to < 18% of community compositional variations among turfgrass systems. Our results indicate that defoliation management was the main factor in shaping the soil microbial community and grass growth habits was secondary in modulating microbial taxon distribution.

## Introduction

Turfgrass, covering over 16 million hectares in the US, is one of the most important irrigated crops in the country and provides significant regulating (e.g., C sequestration, soil erosion control, and cooling), supporting (e.g., nutrient cycles), and cultural (e.g. spiritual and recreational benefits) services [1–3]. Characterized with intensive management, including fertilization, irrigation, and the use of pesticide, however, the mono-cultured turf (i.e., grass and the subtending soil) has long been criticized for the high rate of fertilization and thus feedforward effects on the environment due to nutrient loss. Over years, research emphasis has been on seeking management practices to improve fertilizer use efficiency and mitigate nutrient loss (e.g., N leaching and gas emissions), and also continuously introducing new cultivars to reduce management cost while increasing sustainability [4,5]. There are a number of ways to choose turfgrass species, but all depend on grass species characteristics, e.g., appearance, cultivation requirement, pest resistance, and stress tolerance. While knowledge has been considerably advanced on turfgrass physiology and ecology, information is still lacking on how grass species modulates the diversity, composition, and function of the soil microbial community, the key component for understanding N cycle and thus the fate of N in the environment.

A plant species may exert multiple selection pressures on soil microbes. Rhizodeposits, the root-derived C compounds that originate from sloughed-off of root cells and tissues, mucilages, volatiles, and soluble lysates and exudates [6], are a strong determinant of the soil microbial community. As a readily available and rich C source, rhizodeposits may facilitate the proliferation of copiotrophs over oligotrophs to enhance competition exclusion and therefore reduce microbial species richness and evenness [7–9]. Rhizodeposits may also target specific microbial taxa and thus shape the soil microbial community [10]. Nonetheless, such effects are often spatially restricted to the rhizosphere. Plants also affect microbes in the root zone and bulk soil through controls on soil physical and chemical properties, including pH, nutrients, and pore size and distribution. For instance, roots stimulate soil aggregation and stability through physical entanglement and also through mucilage production to bind soil particles. Roots can alter soil pH via cation-anion exchange balance, organic anion release, root exudation and respiration, and redox-coupled processes [11]. Further, root architecture (e.g., elongation rate, lateral root production, and root length density) may interfere with soil hydraulic conductivity to regulate water flow and nutrient movement [12]. Such plant-driven selection on microbes has been increasingly recognized, and selective effects have been found to differ even at a plant species or genotype scale [12–15].

Turfgrasses vary largely in growth habits, despite that all possess narrow leaves and fibrous roots. Based on ways of new growth generation, turfgrasses can be classified as either tussock grass or non-tussock grass. A tussock grass (or bunch-type grass) produces new grasses from tillers in a cluster or bunch. In contrast, a non-tussock grass (or creeping-type grass) produces new grasses from above- and/or belowground lateral stems (i.e., stolons and rhizomes, respectively). As such, stolonferous and rhizomatous grasses have a greater capacity to spread laterally relative to tussock grasses and can quickly make a dense and uniform land cover. Turfgrasses can also be grouped into C3 cool-season and C4 warm-season grasses, with C4 being more efficient for photosynthesis at elevated temperature and more tolerant to drought, and thus being preferred in warm and arid regions. While some geographic areas are suitable for both warm- and cool-season grasses [16], most cool-season turfgrasses will suffer the loss of root system during mid-summer when soil temperature rises above 17°C [17]. Because grow habits might affect belowground resource (e.g., C, N, and water) availability and distribution, we hypothesized that soil microbial communities would differ between warm- and cool-season grasses and between bunch-type and creeping-type grasses. The main objective of this work was to examine how soil microbial species richness, diversity, and N-cycle functional genes vary with turfgrass species identities and growth habits.

The investigation took advantage of an existing turfgrass site where grasses of different growth habits had been established on soils of similar texture and subjected to different defoliation practices (e.g., mowing intensity and frequency). Defoliation may affect root architecture, morphology, and biomass allocation [18,19] and therefore the assemblage of soil microbes. By including defoliation management, we could better evaluate the impacts of grass growth habits. We also examined relationships of microbial community structural metrics with soil properties, considering the influences of edaphic factors, e.g., pH, moisture, and soil texture on the soil microbial community [20–24].

## Materials and methods

### Field plots and soil sampling

Soil samples were taken from individual plots of six turfgrass species at the Lake Wheeler Turfgrass Field Laboratory, North Carolina State University, Raleigh NC, USA in August 2016. The six species included bermudagrass (*Cynodon dactylon* x *C*. *transvaalensis* cv. ‘Champion’), creeping bentgrass (*Agrostis stolonifera* cv. ‘Penncross’), Kentucky bluegrass (*Poa pratensis*), tall fescue (*Festuca arundinacea*), St. Augustinegrass (*Stenotaphrum secundatum* cv. ‘Raleigh’), and zoysiagrass (*Zoysia japonica* cv. ‘El Toro’) and had been established over 5 years (see Table 1 for grass growth habits and defoliation management). It is worth mentioning that Greens (Table 1) were mown more frequently and at a lower height than Non-greens. The plot sizes of these turfgrass species varied, being smallest, ~ 0.1 ha for St. Augustinegrass (ST), Kentucky bluegrass (KB) and zoysiagrass (ZG), and largest, ~ 0.4 ha for tall fescue (TF). All turfgrasses had been managed for mowing, irrigation, and fertilization according to professional standards. In brief, for the non-putting greens, fertilizers were applied two or three times a year with a cumulative rate of 100–195 kg N ha^-1^ yr^-1^ and mostly in summer for warm-season grasses and in spring and fall for cool season grasses. Common herbicide, either glyphosate (C_3_H_8_NO_5_P) or oxadiazon (C_15_H_18_Cl_2_N_2_O_3_) was applied yearly. The two putting greens, bermudagrass (BM) and creeping bentgrass (CB), had fertilizers applied more frequently throughout the year at a rate of 191 and 333 kg N ha^-1^ yr^-1^, respectively. Fungicides like triadimefon, triticonazole, and fluoxastrobin were also applied to BM and CB with a rate of 50 and 150 kg ha^-1^ yr^-1^, respectively. Irrigation was applied as needed to prevent stress. Soil in the plots is classified as fine sandy loam (fine, kaolinitic, thermic Typic Kanhapludults).

**Table 1:** Growth habits and defoliation management of six turfgrass species. Defoliation was more intensive and frequent in green than in non-green turfgrass systems.

	Photosynthesis	Asexual propagation	Defoliation
Bermudagrass (BM)	Warm-season, C4	Non-tussock	Green
Creeping bentgrass (CB)	Cool-season, C3	Non-tussock	Green
Kentucky bluegrass (KB)	Cool-season, C3	Non-tussock	Non-green
Tall fescue (TF)	Cool-season, C3	Tussock	Non-green
St. Augustinegrass (ST)	Warm-season, C4	Non-tussock	Non-green
Zoysiagrass (ZG)	Warm-season, C4	Non-tussock	Non-green

To make representative samples, each turf plot was further divided into three roughly equal-size subplots. Then, six to eight soil cores (2.5 cm dia. x 10 cm length) were taken randomly from spots of >1 m away from edges of each subplot and mixed, resulting in three composite samples for each grass species. For TF, three composite samples also represented three cultivars, ‘Fesnova’, ‘Raptor III’, and ‘Regenerook’, respectively. There were a total of 18 composite soil samples (i.e., 6 turfgrass species x 3 subplots as replicates). Soil was sieved (< 2 mm), one aliquot stored at -20°C prior to DNA extraction, and the other aliquot stored at 4°C until the analyses of soil and microbial properties.

### Soil chemical and biological properties

Soil pH was determined with a soil (g)/water (ml) ratio of 1:2.5. Dry combustion method was used to analyze soil total C and N with a Perkin-Elmer 2400 CHN analyzer (Perkin-Elmer Corporation, Norwalk, CT, USA). Soil inorganic N (NH_4_^+^-N and NO_3_^—^N) was extracted using 0.5M K_2_SO_4_ at a ratio of 1:5 soil (g)/solution (ml), filtered through Whatman #42 filter paper, and determined with FIA QuikChem 8000 autoanalyzer (Lachat Instruments, Loveland, CO, USA). Extracted total C and N in solution were also analyzed with TOC analyzer (TOC-5000, Shimadzu Scientific Instruments, Japan). A chloroform fumigation-extraction method was used to determine soil microbial biomass C (MBC) and N (MBN) with extraction coefficients of 0.38 and 0.54 to biomass C and N, respectively [25,26].

### DNA extraction, amplification and sequencing

Soil DNA was extracted using FastDNA Spin Kit for Soil (MP Bio, Solon, OH, USA) from 0.6 g of each soil sample. The extracted DNA was then column-purified using OneStep PCR Inhibitor Removal Kit (Zymo Research, Orang, CA, USA) and a concentration of more than 50 ng μL^-1^ was acquired for each sample. DNA purity of about 1.70–1.90 was assessed by the ratio of absorbance at 260 and 280 nm using a NanoDrop Spectrophotometer (Thermo Scientific, Wilmington, DE, USA).

Bacterial 16S rRNA gene and fungal ITS was PCR-amplified with primer pairs targeting V3-V4 (F319: 5’-ACTCCTACGGGAGGCAGCAG-3’ and R806: 5’- GGACTACHVGGGTWTCTAAT-3’) and ITS1-ITS2 (F_KYO2: 5’-TAGAGGAAGTAAAAGTCGTAA-3’ and R_KYO2: 5’- TTYRCTRCGTTCTTCATC-3’), respectively, and with Illumina MiSeq overhang adapters [27,28]. The PCR was a 50 μL reaction consisting of 25 μL 2x KAPA HiFi HotStart ReadyMix (KAPA Biosystems, Wilmington, MA, USA), 2.5 μL template DNA (4–20 ng μL^-1^), 2.5 μL 10 mM of each primer, and 17.5 μL nuclease-free water. A negative control with no DNA template was also included as a control of extraneous DNA contamination. The thermocycling condition for PCR of 16S rRNA genes was: initial denaturation at 95 ^o^C for 3 min; 25 cycles of 98 ^o^C for 30 sec, 55 ^o^C for 15 sec, and 72 ^o^C for 30 sec; final elongation at 72 ^o^C for 5 min. For fungi, all the thermocycling condition was the same except for that annealing temperature was 51 ^o^C. PCR products were cleaned up with AMPure XP beads (Beckman Coulter Genomics, Danvers, MA, USA) and then eluted in 10 mM Tris buffer (pH 8.5). Unique index (barcode) sequences were added to purified DNA fragments at both ends using the Nextera XT Index Kit (Illumina, San Diego, CA, USA) and a second clean-up was performed. The purified 16S rRNA gene and ITS fragments were mixed equimolarly and paired-end sequenced on Illumina Miseq platform (300×2 paired end, v3 chemistry) (Illumina, San Diego, CA, USA). Due to no detectable DNA amplification, negative controls were not sequenced. The Miseq sequences were deposited in GenBank with the BioProject accession number PRJNA484409.

### Bioinformatics analysis

Demultiplexed sequencing data were trimmed based on the expectation of amplicon size (430-470bp for 16S rRNA gene and 180-360bp for ITS), filtered by the maximum error rate, 0.5% using USEARCH v9.1.13 [29], and then chimeras of ~ 40% sequence reads on average identified and removed using a usearch61 method in QIIME 1.9.1 [30]. Operational taxonomic units (OTUs) were classified with a threshold of 97% similarity and then assigned to taxa using the open reference algorithm for both 16S rRNA gene and ITS sequencing data by using methods of usearch61 against Greengenes database (13_8) and RDP (Ribosomal Database Project) against UNITE database (12_11) [31–33], respectively. Singletons were removed during OTUs pickup; and sequences in a range of ~ 30,000 to 300,000 across samples were rarefied to a depth of 27,000 and 25,000 for bacteria and fungi, respectively. Alpha diversity metrics, including observed OTUs based on rarefaction curves, chao1, and Shannon index were analyzed in QIIME. Matrices of weighted unifrac and Bray-Curtis distance were used for beta diversity analysis of bacterial and fungal communities, respectively, by principal coordinate analysis (PCoA).

Relative abundance of putative functional genes involved in N cycle was predicted using PICRUSt (Phylogenetic Investigation of Communities by Reconstruction of Unobserved States) [34] by matching bacterial OTUs predicted from 16S rRNA gene with reference genomes. Bacterial OTUs with genes involved in N cycle processes were collected using metagenome_contributions.py script based on Kyoto Encyclopedia of Genes and Genomes (KEGG) orthology (KO) database and were then taxonomically classified. Generally, an NSTI (i.e., weighted nearest sequenced taxon index) of around 0.17 or less was considered reliable for soil samples [34]. The NSTI scores of our samples were 0.18 ± 0.01 (standard deviation).

### Statistical analyses

One-way ANOVA, followed by post-hoc Tukey’s test (SAS 9.4, SAS Institute Inc. Cary, NC, USA) was performed for multiple comparisons of soil biochemical properties, microbial alpha diversity, and N-cycle gene relative abundances among grass species. Correlations between soil biochemical properties and microbial communities were analyzed using DistLM (distance-based linear models) in PRIMER (Plymouth Routines in Multivariate Ecological Research Statistical Software, v7.0.13, PRIMER-E Ltd, UK); forward selection was applied to add one soil property to the model at each step and the property increasing adjusted R^2^ most at each step was chosen. All the statistical significance was based on *P* ≤ 0.05 if not specified. Significance of sample grouping in PCoA was analyzed using Adonis method in QIIME. Significance of taxon relative abundance was determined using linear discriminant analysis effect size (LEfSe) (http://huttenhower.sph.harvard.edu/galaxy/) [35].

## Results

### Bacterial and fungal communities

**Fig 1:**
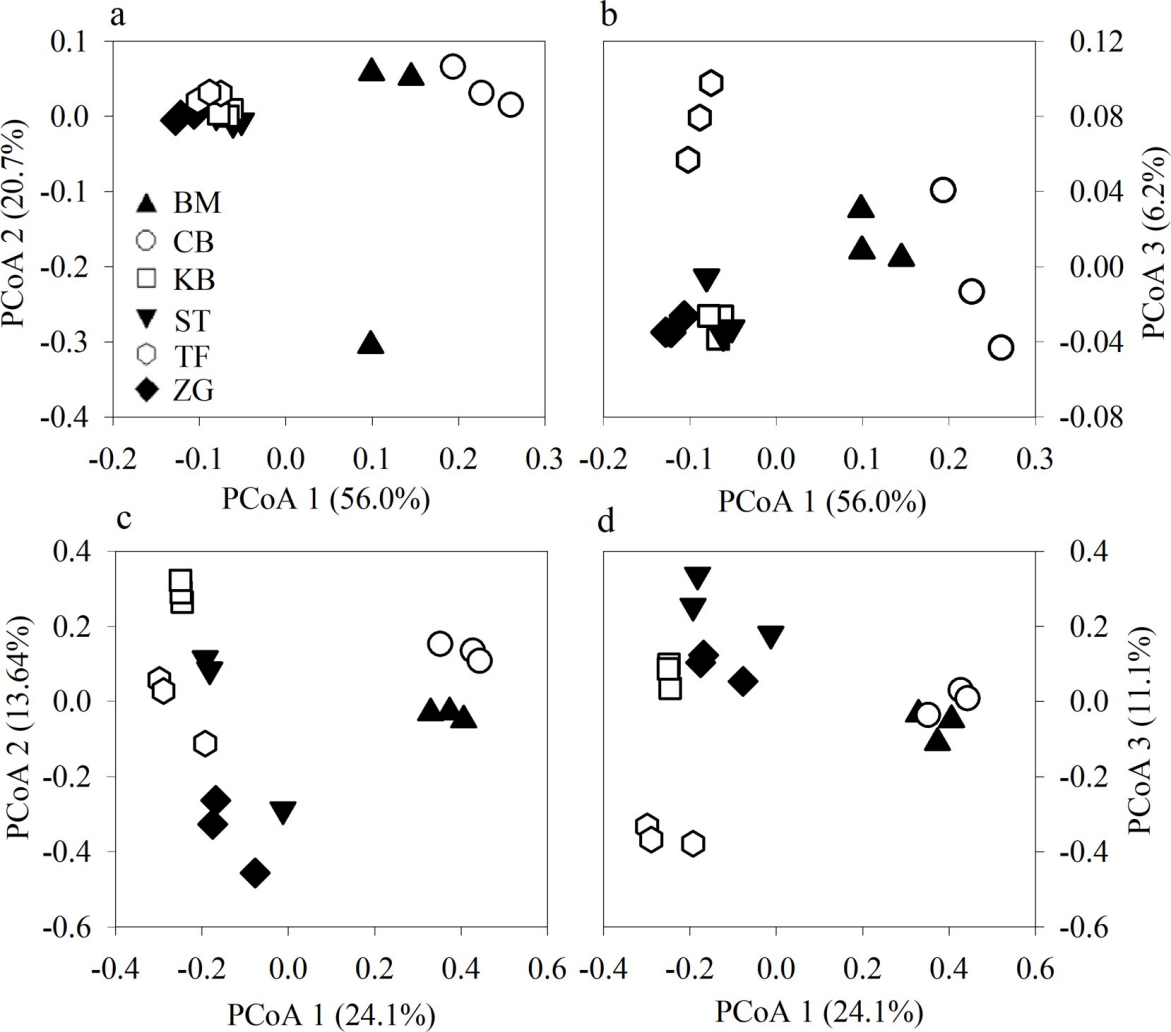
Principal coordinate analysis (PCoA) of soil microbial communities. Soil bacterial (a, b) and fungal (c, d) communities in six turfgrass systems (BM, bermudagrass; CB, creeping bentgrass; KB, Kentucky bluegrass; TF, tall fescue; ST, St. Augustinegrass; ZG, zoysiagrass). Cool- and warm-season turfgrass systems are represented by empty and filled symbols, respectively.

Both bacterial and fungal species richness (i.e. observed OTUs and chao1) were grass species-dependent, being greatest in tall fescue, following by zoysiagrass, St. Augustine, Kentucky bluegrass, and bermudagrass, and lowest in creeping bentgrass (Table 2). Shannon diversity index followed the similar pattern except for the index for the fungal community of creeping bentgrass. However, neither microbial richness nor Shannon diversity index differed between warm- and cool- season grass species. PCoA analysis showed differences in the soil microbial community structure among grass species, with bermudagrass and creeping bentgrass of more intensive defoliation management being similar (Fig 1). Minor and yet significant differences were also found between tall fescue and other grass species along the PCoA-3, and between warm- and cool-season grasses for the fungal community along the PCoA-2.

**Table 2:** Soil microbial alpha diversity metrics (means ± standard errors) in six turfgrass systems (BM, bermudagrass; CB, creeping bentgrass; KB, Kentucky bluegrass; TF, tall fescue; ST, St. Augustinegrass; ZG, zoysiagrass), estimated from a sequence depth of 27,000 for bacteria and 25,000 for fungi. Different letters within each row indicate significant differences at *P* < 0.05.

	Cool-season (C3) grasses			Warm-season (C4) grasses		
	CB	KB	TF	BM	ST	ZG
Bacteria						
Observed OTUs	4622±222 d	6263±32 c	7059±107 a	6152±797 bc	6626±37 b	6645±108 b
Chao1	8030±541 d	11637±68 c	13764±396 a	11624±1562 bc	12588±117 b	13057±394 ab
Shannon index	10.1±0.2 e	11.2±0.0 c	11.4±0.0 a	11.0±0.3 bc	11.3±0.0 b	11.3±0.0 b
Fungi						
Observed OTUs	1094±60 c	1122±35bc	1391±5 a	1136±81 bc	1154±152 bc	1169±73 b
Chao1	1303±58 e	1488±45 d	1714±29 a	1553±85 cd	1664±170 ab	1604±155 bc
Shannon index	7.4±0.1 a	6.9±0.0 c	7.5±0.1 a	6.6±0.2 d	6.5±0.4 d	7.2±0.1 b

**Fig 2:**
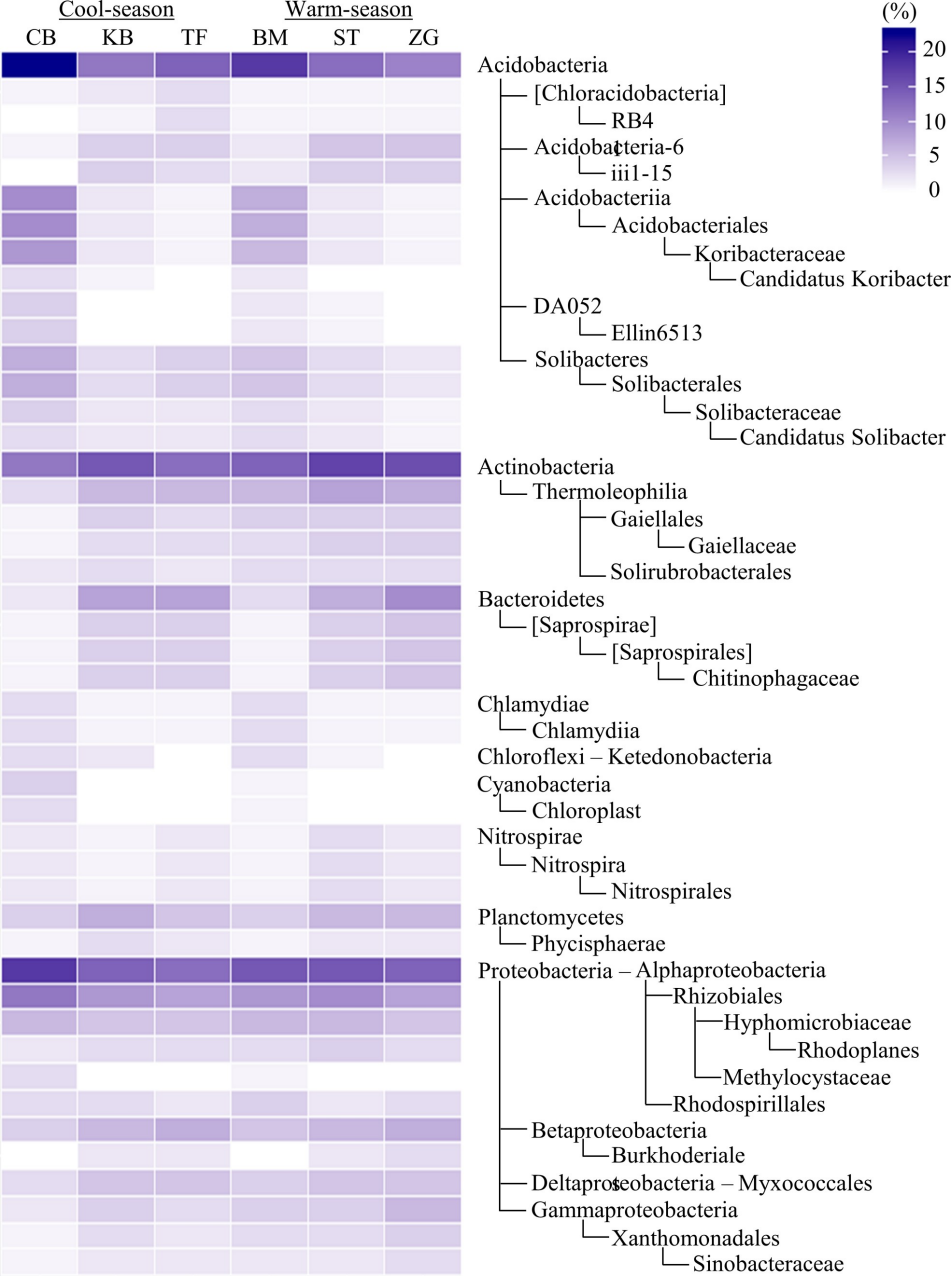
The heatmap of soil bacterial taxa. The heatmap contained bacterial taxa that differed significantly among six turfgrass systems (BM, bermudagrass; CB, creeping bentgrass; KB, Kentucky bluegrass; TF, tall fescue; ST, St. Augustinegrass; ZG, zoysiagrass). Only taxa with ≥ 2.5% relative abundance and assigned at least to the phylum level are included. The color scale indicates the relative abundance (%).

A total of 47 bacterial phyla were detected in turfgrass systems, while only 13 had a relative abundance > 1% (S1 Fig). The most abundant phylum was Proteobacteria, accounting for ~32%, followed by Acidobacteria (~15%), Actinobacteria (~14%) and Chloroflexi (~8%). Of 269 identified bacterial taxa from phyla to genera with > 0.1% relative abundance, ~77% differed significantly among turfgrass systems (Fig 2). Compared to other grass species, creeping bentgrass was most abundant in Acidobacteria and Alphaproteobacteria, and least in Actinobacteria, Bacteroidetes and Planctomycetes. In contrast, St. Augustinegrass was relatively more abundant in Actinobacteria; zoysiagrass in Bacteroidetes; and Kentucky bluegrass in Planctomycetes. There were also compositional differences among grass species in the sublevel taxa from class to genus. For example, [Chloracidobacteria] was relatively abundant in in tall fescue; Rhodospirillales in bermudagrass; and Acidobacteriales, an order of Acidobacteria, accounting for 10% in creeping bentgrass. Similarity between creeping bentgrass and bermudagrass in Fig 1 was in line with similarity in the relative abundances of taxonomic groups. They both showed greater abundance in Acidobacteria, Chlamydiae, Cyanobacteria, Hyphomicrobiaceae, and Methylocystaceae, but lower abundance in Bacteroidetes, Planctomycetes, and Betaproteobacteria, compared to other grasses. Minor and yet significant differences in microbial community compositions were also associated with cool- vs. warm-season grasses, with cool-season grasses being relatively more abundant in Chlorofexi and Verrucomicrobia (S2 Fig).

**Fig 3:**
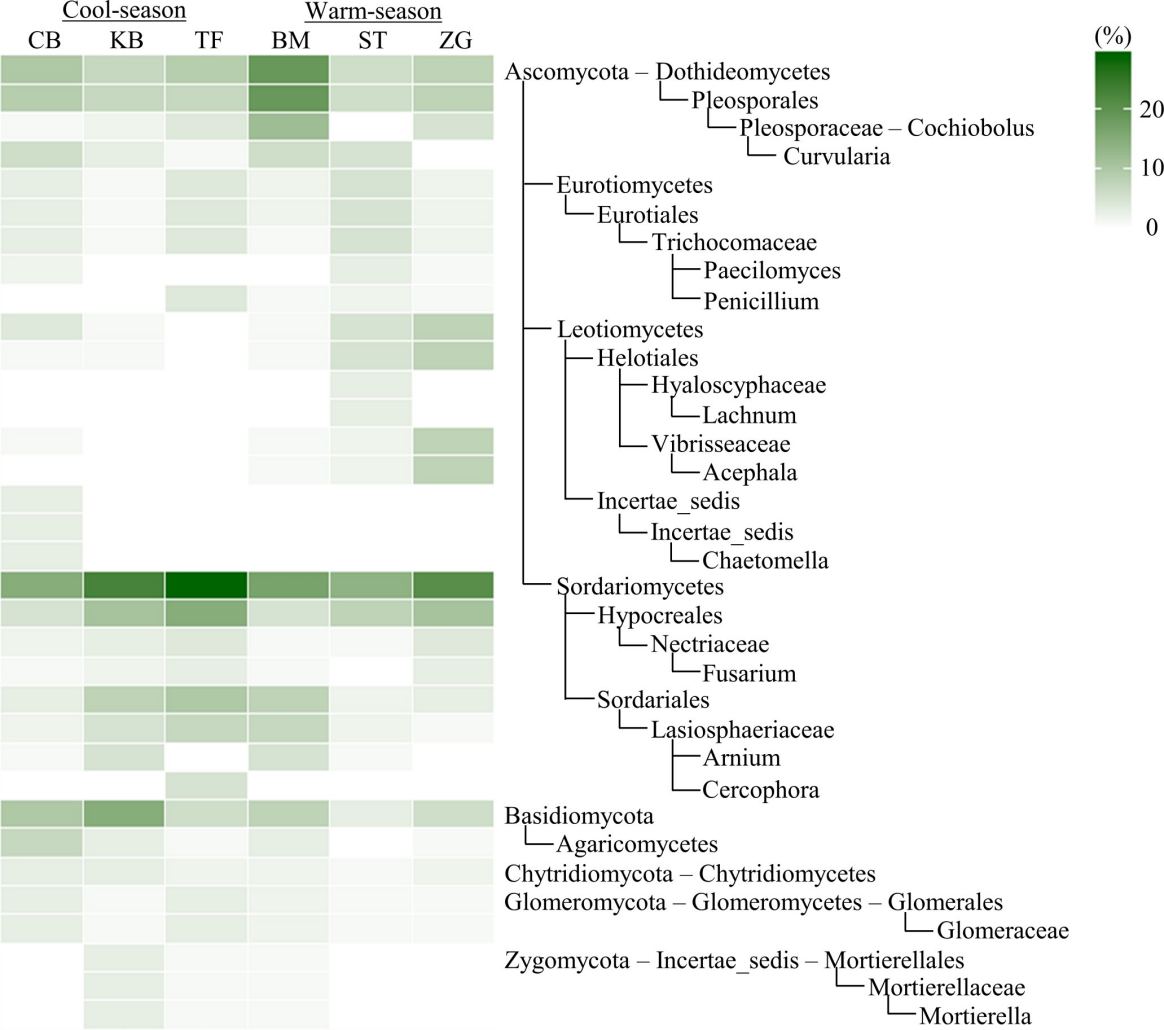
The heatmap of soil fungal taxa. The heatmap contained fungal taxa that differed significantly among six turfgrass systems (BM, bermudagrass; CB, creeping bentgrass; KB, Kentucky bluegrass; TF, tall fescue; ST, St. Augustinegrass; ZG, zoysiagrass). Only taxa with ≥ 2.5% relative abundance and assigned at least to the phylum level are included. The color scale indicates the relative abundance (%).

Unlike bacteria, a great portion of ITS sequences (~ 35%) could not be assigned into a phylum and lower taxonomic ranks, except into the fungal domain (S1 Fig), perhaps because of incompleteness of the UNITE database and/or non-target amplicons of other eukaryotes. This portion was considered to have little impact on fungal community comparisons since its proportion was similar among samples. Nonetheless, turfgrasses were dominant with Ascomycota (~ 44% on average across six turfgrass systems), followed by Basidiomycota (~ 8%). Of 111 identified taxa with > 0.1% abundance, ~ 78% showed significant differences among turfgrass systems. Basidiomycota and Zygomycota were most abundant in Kentucky bluegrass, accounting for 15.2% and 2.6%, respectively (Fig 3). Sordariomycetes, the class of Ascomycota was relatively more abundant in tall fescue, whereas Dothideomycetes, Eurotiomycetes, and Leotiomycetes were relatively more abundant in warm-season grasses (bermudagrass, St. Augustinegrass, and zoysiagrass). Some taxa presented solely in one turfgrass system but negligible in all the others, e.g., Incertae_sedis accounting for 3.4% in creeping bentgrass while < 0.1% in the other five turfgrass systems. Moderate differences in taxa were also found between cool- and warm- season grasses, with Chytridiomycota and Glomeromycota relatively more abundant in cool-season grasses (S2 Fig).

### Gene abundances involved in N cycling

**Fig 4:**
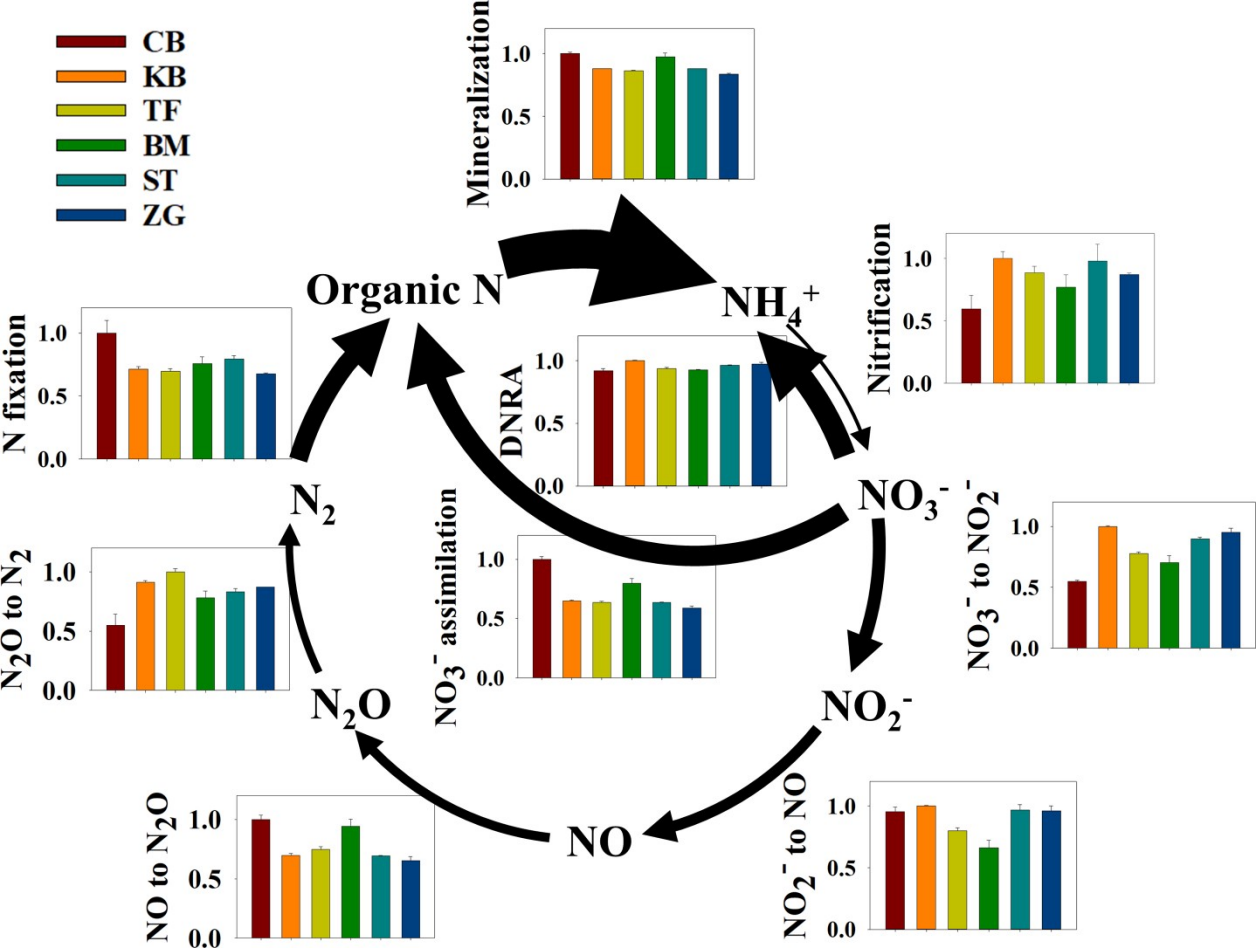
Relative abundances of genes involved in soil N processes. Genes for N transformations were predicted from the bacterial marker gene 16S rRNA using PICRUSt with a sequence depth of 19,890. Arrow thickness is positively related to gene abundances of individual N pathways, and bar height reflects relative gene abundances, which were nomalized to the highest values of indivisual processes among six turfgrass systems (BM, bermudagrass; CB, creeping bentgrass; KB, Kentucky bluegrass; TF, tall fescue; ST, St. Augustinegrass; ZG, zoysiagrass). Gene abundances were calculated as: K00260 + K00261 + K00262 for mineralization; ((K10944 + K10945 + K10946)/3 + K10535)/2 for nitrification; (((K00370 + K00371 + K00374 + K00373)/4 + (K02567 + K02568)/2) + (K00362 + K00363)/2 + K03385)/2 for dissimilatory NO_3_^-^ reduction to NH_4_^+^ (DNRA); (K00367 + K00372 + K00360 + K00366)/2 for assimilatory NO_3_^-^ reduction to NH_4_^+^; (K02588+K02586+K02591)/3+K00531 for N fixation; (K00370+K00371+K00374+K00373)/4 +(K02567+K02568)/2 for dissimilatory NO_3_^-^ reduction to NO_2_^-^; K00368 for dissimilatory NO_2_^-^ reduction to NO; (K04561+K02305)/2 for dissimilatory NO reduction to N_2_O; K00376 for dissimilatory N_2_O reduction to N_2_.

Relative abundances of genes involved in N cycle varied significantly with specific pathways, being the greatest for mineralization, followed by assimilatory NO_3_^-^ reduction, dissimilatory nitrate reduction to ammonium (DNRA), N fixation, denitrification, and the lowest for nitrification (Fig 4). However, there were significant variations in all N cycle processes among turfgrasses. Creeping bentgrass showed the lowest relative abundance of nitrification gene but the greatest relative abundances of genes in assimilatory NO_3_^-^ reduction and N fixation. In addition, this turfgrass system was characterized by the lowest relative gene abundances in dissimilatory NO_3_^-^ reduction to NO_2_^-^ and N_2_O reduction to N_2_. As a result, the ratio of relative gene abundances between mineralization and nitrification in creeping bentgrass was nearly two-fold greater than that of other turfgrasses except for bermudagrass. However, creeping bentgrass had the lowest ratio of nitrification to assimilatory NO_3_^-^ reduction and the lowest ratio of dissimilatory N_2_O reduction to dissimilatory NO reduction.

OTUs possessing these genes also diverged among turfgrasses, showing similar patterns to those of the total bacterial community. Dominant phyla involved in *nosZ* for dissimilatory N_2_O reduction to N_2_ were Proteobacteria and Chloroflexi, whereas dominant phyla in *hao* for hydroxylamine hydrolysis were Proteobacteria, Plantomycetes and Nitrospriae (S3 Fig). Compared to other grasses, creeping bentgrass had the lower relative abundance of *hao* in Plantomycetes but more in Proteobacteria. This turfgrass system also had lower *nosZ* abundance in Bacteriodetes but more in Chloroflexi.

### Correlations of microbial community attributes with soil properties

Soil properties differed significantly among turfgrasses, but coefficients of variation were moderate, being lowest for pH, ~7.4% and highest for soil organic N ~ 44% (Table 3). All the properties did not show significant differences between cool- and warm-season grasses.

Distance based linear model analysis showed that only soil pH was significantly correlated with both bacterial and fungal community compositions, explaining ~ 18% and 12% of the total variations, respectively (Table 4). Several other soil properties were also significantly correlated with fungal community compositions, and yet all explained no more than 10% of the total variation. Soil pH, extractable organic C, and inorganic N together explained 43% of the total variation in the bacterial community; and soil pH, NH_4_^+^, and extractable organic C together explained ~34% of total variation in the fungal community (Table 5).

**Table 3:** Selected soil properties (means ± standard errors) in six turfgrass systems (BM, bermudagrass; CB, creeping bentgrass; KB, Kentucky bluegrass; TF, tall fescue; ST, St. Augustinegrass; ZG, zoysiagrass). Different letters within each row indicate significant differences at *P* < 0.05.

	Cool-season (C3) grasses			Warm-season (C4) grasses		
	CB	KB	TF	BM	ST	ZG
Soil pH	4.99±0.17bc	5.16±0.03b	5.01±0.03bc	4.73±0.02c	4.95±0.02bc	5.82±0.02a
Soil C (g kg^-1^ soil)	18.9±1.3b	9.3±0.4c	20.0±1.7ab	14.0±0.2bc	14.2±2.1bc	27.2±2.4a
Soil N (g kg^-1^ soil)	1.5±0.1b	0.6±0.0d	1.4±0.1bc	1.0±0.0bcd	0.9±0.1cd	2.2±0.2a
MBC (mg kg^-1^ soil)	297±15ab	260±30b	255±24b	212±20b	280±57ab	457±58a
MBN (mg kg^-1^ soil)	70.6±4.9b	37.3±5.3c	61.0±4.6bc	52.6±4.9bc	54.5±1.2bc	98.8±8.8a
MBC:MBN	4.2±0.1b	7.0±0.4a	4.2±0.1b	4.0±0.0b	5.1+1.0ab	4.6±0.3b
NH_4_^+^-N (mg kg^-1^ soil)	11.1±0.1b	5.0±0.7c	11.1±0.4b	10.1±0.9b	6.5±0.6c	15.1±1.1a
NO_3_^—^N (mg kg^-1^ soil)	3.5±0.1ab	1.8±0.1b	4.1±0.0ab	2.8±0.7ab	1.8±0.1b	4.8±1.0a
Inorganic N (mg kg^-1^ soil)	14.6±0.1b	6.8±0.5c	15.2±0.4b	12.9±1.2b	8.3±0.6c	19.8±0.6a
Extractable organic N (mg kg^-1^ soil)	9.1±0.3ab	9.5±1.0a	6.4±0.7bc	6.2±0.5c	8.4±0.6abc	10.8±0.2a
Extractable organic C (mg kg^-1^ soil)	86.3±0.4ab	84.6±5.4ab	65.2±0.4c	65.8±3.1c	73.6±1.5bc	96.1±2.1a

**Table 4:** Results of marginal tests by DistLM.

Variable	SS(Trace)	Pseudo-F	P value	Proportion
Bacteria				
Soil pH	918.7	3.44	0.021	0.177
Soil C	207.8	0.67	0.573	0.040
Soil N	167.9	0.54	0.683	0.032
MBC	373.0	1.24	0.280	0.072
MBN	180.9	0.58	0.637	0.035
MBC:MBN	474.7	1.61	0.172	0.091
NH_4_^+^	161.3	0.51	0.713	0.031
NO_3_^-^	177.3	0.57	0.635	0.034
Inorganic N	165.8	0.53	0.719	0.032
Organic N	340.4	1.12	0.306	0.066
Extractable C	243.3	0.79	0.496	0.047
Moisture	461.2	1.56	0.203	0.089
Fungi				
Soil pH	7179	2.17	0.010	0.119
Soil C	5923	1.75	0.038	0.099
Soil N	5921	1.75	0.036	0.098
MBC	5712	1.68	0.045	0.095
MBN	5986	1.77	0.031	0.100
MBC:MBN	6097	1.81	0.029	0.101
NH_4_^+^	6304	1.87	0.026	0.105
NO_3_^-^	5161	1.50	0.085	0.086
Inorganic N	6363	1.89	0.028	0.106
Organic N	5185	1.51	0.086	0.086
Extractable C	4834	1.40	0.125	0.080
Moisture	2974	0.83	0.702	0.049

**Table 5:** Results of sequential tests with forward selection by DistLM.

Variable	R^2^	SS (trace)	Pseudo-F	P value	Proportion	Cumulation
Bacteria						
Soil pH	0.126	918.7	3.44	0.023	0.177	0.177
Extractable C	0.227	728.2	3.09	0.029	0.140	0.318
Inorganic N	0.309	589.9	2.80	0.031	0.114	0.431
Moisture	0.349	368.2	1.85	0.141	0.071	0.502
Soil C	0.369	269.7	1.40	0.234	0.052	0.554
Soil N	0.380	231.0	1.22	0.294	0.045	0.599
Fungi						
Soil pH	0.064	7179	2.17	0.009	0.119	0.119
NH_4_^+^	0.142	7340	2.44	0.005	0.123	0.243
Extractable C	0.195	5683	2.00	0.015	0.095	0.337
Soil C	0.211	3597	1.29	0.190	0.060	0.397
MBC:MBN	0.226	3425	1.25	0.239	0.057	0.454
MBC	0.249	3602	1.36	0.185	0.060	0.514
Moisture	0.264	3200	1.23	0.269	0.053	0.567
Soil N	0.274	2920	1.14	0.330	0.049	0.616

## Discussion

Our initial hypothesis was that grass growth habits, both propagation types and photosynthetic pathways, could play important roles in modulating the diversity, composition, and functional gene abundances of the soil microbial community. However, our data showed that propagation types and photosynthetic pathways affected different metrics of the soil microbial community. Propagation types appeared to regulate microbial species richness, whereas photosynthetic pathways controlled the community composition despite moderate influences compared to defoliation management.

### Propagation type affected the alpha diversity of the microbial community

Both microbial species richness and Shannon diversity index were greatest in tall fescue, but no single examined soil property could explain such variations. Instead, grass growth habit seemed to be the cause. Of the six grasses, tall fescue was the only tussock-type grass. Due to tiller production and no lateral stems, this grass grows as singular plants in tufts, leading to uneven soil coverage of shoots and roots. In contrast, non-tussock grasses generated new growth by aboveground stolons and/or belowground rhizomes and were likely to form a uniform lawn.

Resource translocation and information sharing among ramets of clonal plants have been well studied [36]. Ramets can translocate water, carbohydrates, and minerals from individuals with high supply to those with low supply. Plants may also share information by translocating signal molecules [37,38] or secreting massive perfumes [39] when they are exposed to herbivore damage and defoliation. It is possible that chemical and information sharing among ramets promoted physiological synchronization among individuals and thus enhanced similarity in rhizodeposit biochemistry. Relatively ‘long-distant’ ramets of non-tussock grasses might distribute C, N, water, and other resources over a wide spatial scale and helped to increase resource homogeneity. In contrast, ramets of tussock grasses were clustered, resulting in resource distribution in a more confined area. As such, tussock grasses were more likely to introduce a fine-scale heterogeneity in soil properties.

Fine-scale heterogeneity in soil physicochemical properties (e.g. organic C, nutrients, water, pH, and aerobic conditions) has been considered as a key driver to promote biodiversity [40,41]. Often, fine-scale heterogeneity in soil is realized through aggregation [42,43], because not only does it help to create more divergent niches for species adaptation, but also it helps to separate competitive species and thereby limit competitive exclusion [44–46]. Accordingly, factors that influence aggregation are expected to impact soil biodiversity. Although soil organic matter could positively contribute to soil aggregation [47,48] and varied up to three fold among grass species in this work, it did not contribute to the divergence in microbial species richness. This concurred with another work where microbial species richness was found to be stable over a chronosequence of bermudagrass systems, despite ~ three-fold differences in soil organic C [49]. Together, these suggest that root type rather than soil organic C content was the main driver for the divergence of microbial species richness among turfgrass systems.

Our data have two implications. First, growth habit-associated fibrous roots might contribute largely to divergence in soil aggregation. Second, non-tussock grasses might promote resource translocation and information sharing, and thereby improving the fine-scale homogeneity of soil properties. Hence, soil cultivated with stoloniferous or rhizomatous grass species was prone to harbor the microbial community of lower richness, compared with soil cultivated with the tussock-type tall fescue.

### Growth habit and defoliation intensity shaped the soil microbial community

As hypothesized, growth habits did help to structure the soil microbial community. However, their effects were moderate, given that tussock-type tall fescue differed from other grasses mainly along the PCoA-3, and the warm-season grasses differed from the cool-season grasses only for fungi along the PCoA-2. Compared to the warm-season grasses, cool-season grasses enhanced the proliferation of Chloroflexi, Verrucomicrobia, Chytridiomycota, and Glomeromycota, the phyla that have been documented to be sensitive to soil nutrient status and/or play roles in plant nutrient uptake [50–52]. In summer, root dieback occurred for cool-season grasses when their photosynthetic activities were reduced by high temperature and yet they were still actively respiring. Because grass demanded more carbohydrates than photosynthesis could provide, root mass would decrease dramatically. As a consequence, grass ability of water and nutrient uptake declined. Our results suggest that to meet the challenge of root mass loss in summer, cool-season grasses might recruit beneficial microbes to help water and nutrient uptake.

Soil microbial communities differed mainly by defoliation intensity since bermudagrass and creeping bentgrass, the two grasses that were mowed at a lower height but greater frequency, clustered and were well separated from other grass species. Several studies examined the impacts of defoliation on the soil microbial community; however, results are mixed [51,53–56]. For example, mowing in a steppe ecosystem had little effect on the soil bacterial community [51], but mowing in a grassland of the Great Plains of North America was found to change the abundance of some bacteria and fungi, e.g., Actinobacteria, Bacteroidetes, Chloroflexi, Planctomycetes, and Ascomycota [56]. Such inconsistency is perhaps because defoliation impacts varied not only with the intensity and frequency of defoliation, but also with the time when evaluations were made (e.g., evaluation right after one-time defoliation versus after years of defoliation). Nonetheless, research tends to support that long-term and cumulative effects of defoliation can be substantial [56,57]. Our results were aligned with those of Bartlett et al. (2008) that soil microbial communities differed between turfgrasses subjected to different intensities of long-term defoliation and management.

Decline in plant photosynthesis and therefore C supply to soil is one of the possible consequences of mowing. Compared to other grasses, more intensive and frequent mowing in bermudagrass and creeping bentgrass might result in a greater reduction of C flow from plant to soil. As such, long-term and intensive defoliation could lead to a resource-poor environment that favored the growth and proliferation of oligotrophic microbes. Indeed, the intensively mowed bermudagrass and creeping bentgrass harbored more Acidobaceria and Alphaproteobacteria but less Betaproteobacteria and Bacteroidetes than other grasses, the former two being documented as oligotrophs and the latter two as copiotrophs [58,59]. Guo et al. (2018) also showed that the relative abundance of Bacteroidetes was significantly reduced after long-term clipping. They also found that genes involved in the decomposition of complex compounds, e.g., starch, hemicellulose, pectin, cellulose, chitin and lignin, were much greater in mowed grassland than in the grassland without defoliation. Together, these results imply that defoliation affected the soil microbial community through controls on the quantity and biochemistry of carbon allocated from plant to soil.

It was reasonable to assume that defoliation management could affect the soil organic C content/biochemistry, but there was no correlation between the two. Neither total soil organic C nor extractable organic C could explain defoliation intensity-based groupings in the soil microbial community. Similar to other studies [21,60], soil pH was the most robust factor in explaining variations of the soil microbial community. However, the explanatory power of pH was small, only accounting for < 18% of total variations in bacteria or fungi. Perhaps, all the soil properties examined in this work were not the most and direct consequences of defoliation management.

Intensively mowed grasses generally demand more additional management practices, such as higher rates of fertilization and pesticide use. A bulk of publications [49,61–63] suggests that plant protection management only played minor roles in shaping the community structure. In this study, N fertilization rates were also unlikely the cause for divergences in the soil microbial community between intensively mowed bermudagrass and creeping bentgrass and other grasses. Nitrogen fertilization in bermudagrass was ~ 40% lower than that in creeping bentgrass but similar to the other grasses. However, microbial communities in bermudagrass and creeping bentgrass were similar and differed from the others. This further helps to infer that defoliation management was a dominant management practice in structuring the soil microbial community.

### Grass species-specific characteristics in N cycle

Nitrogen cycle is the foundation of soil fertility and also crucial to understand the environmental fate of N. A survey of genes encoding enzymes for various N transformations may help to diagnose the potential of soil N supply and efflux at a given ecosystem. Similar to the community structure, N-cycle gene relative abundances drew a clear line between intensively mowed grass systems (creeping bentgrass and bermudagrass) and others. Creeping bentgrass and bermudagrass were characterized by greater relative gene abundances in mineralization, N fixation, and assimilatory nitrate reduction, but lower relative gene abundances in nitrification. Such a N-cycle pattern suggests that the two turfgrass systems were more N limited, and therefore they must mine N through mineralization and N fixation and, on the other hand, reduced the activity that could potentially lead to N loss. Defoliation is known to influence belowground microbial processes, but its magnitude depends on plant species as well as defoliation intensity [53,64]. Generally, defoliation is thought to enhance N mineralization by a pulse input of short-lived labile C [65,66]. Here, we proposed that ephemeral and yet frequent inputs of rhizodeposits in intensively mowed turfgrasses could stimulate microbial activity and turnover. The greater the microbial biomass turnover rate, the more N would be released from microbes for grass uptake. Overtime, a large fraction of N would be locked into grass biomass and removed from soil by defoliation. As a consequent, this would generate a limitation of N to soil microbes despite that N fertilization rates were much greater in creeping bentgrass than the other systems. To combat N limitation, the soil microbial community needed to not only enhance its capacity of N fixation and uptake but also minimize nitrification. Denitrification is another important microbial process that will lead to N loss via gas emissions. The relative abundance of gene involved in the first step (i.e., NO_3_^-^ reduction to NO_2_^-^) of sequential reactions of denitrification was also lower in intensively mowed grasses than the other grasses. This further suggests that defoliation might reduce the processes that lead to soil N loss.

Similar variations in the soil microbial community structure and N cycle gene relative abundances suggest a tight linkage between the community structure and function in turfgrass systems. Nitrogen-cycle gene relative abundances also appeared to correlate with the alpha diversity of the soil microbial community; the lower taxon richness in intensively mowed grasses coincided with the less relative abundance of nitrification genes. Although linkage between microbial community structure and function is often found to be weak [67], loss in microbial diversity has been documented to affect N transformations in soil [68]. Schimel [69] stated whether or not microbial community structure is an important control on ecological processes was the issue of scale; and linkage between microbial community structure and function became loose as the scale was moved up. Nonetheless, our data suggest that mono-cultured turfgrass systems seem to be at the scale that microbial community structure could be used to predict soil functions.

## Conclusions

Grass growth habits (propagation types and photosynthetic pathways) significantly affected soil microbial communities. The tussock-type tall fescue was more beneficial to bacterial and fungal taxon richness than non-tussock grasses, likely due to promotion of soil heterogeneity. Cool-season grasses enhanced the relative abundance of Chloroflexi, Verrucomicrobia, and Glomeromycota, compared to the warm-season grasses, perhaps because as a compensation strategy, root dieback in summer triggered cool-season grasses to recruit microbes for helping nutrient acquisition. However, defoliation intensity was found to be most robust in modulating the soil microbial community and N-cycling gene abundances, with more intensively and frequently mowed turfgrass systems having the lower relative abundances of nitrification genes. This work is significant because it helps to better understand the consequences of the choice of grass species and defoliation management on soil N processes and thus the environmental fate of N.

## Supporting information

S1 Fig Stacked bar charts of microbial community composition. (DOCX)

S2 Fig LEfSe of cool- and warm-season turfgrass systems. (DOCX)

S3 Fig Relative abundances of soil bacterial phyla having *nosZ* and *hao* genes. (DOCX)
