# Supplementary material for: Defoliation management and grass growth habits modulated the soil microbial community of turfgrass systems
Source: PLoS One. 2019 Jun 24;14(6):e0218967. doi: 10.1371/journal.pone.0218967 (PMC6590823; doi:10.1371/journal.pone.0218967)

**S1 Fig. Stacked bar charts of microbial community composition.**

Major soil bacterial (a) and fungal (b) phyla (≥ 1% relative abundances) in six turfgrass systems (see Table 1 for abbreviations) and in cool- and warm-season turfgrass systems.


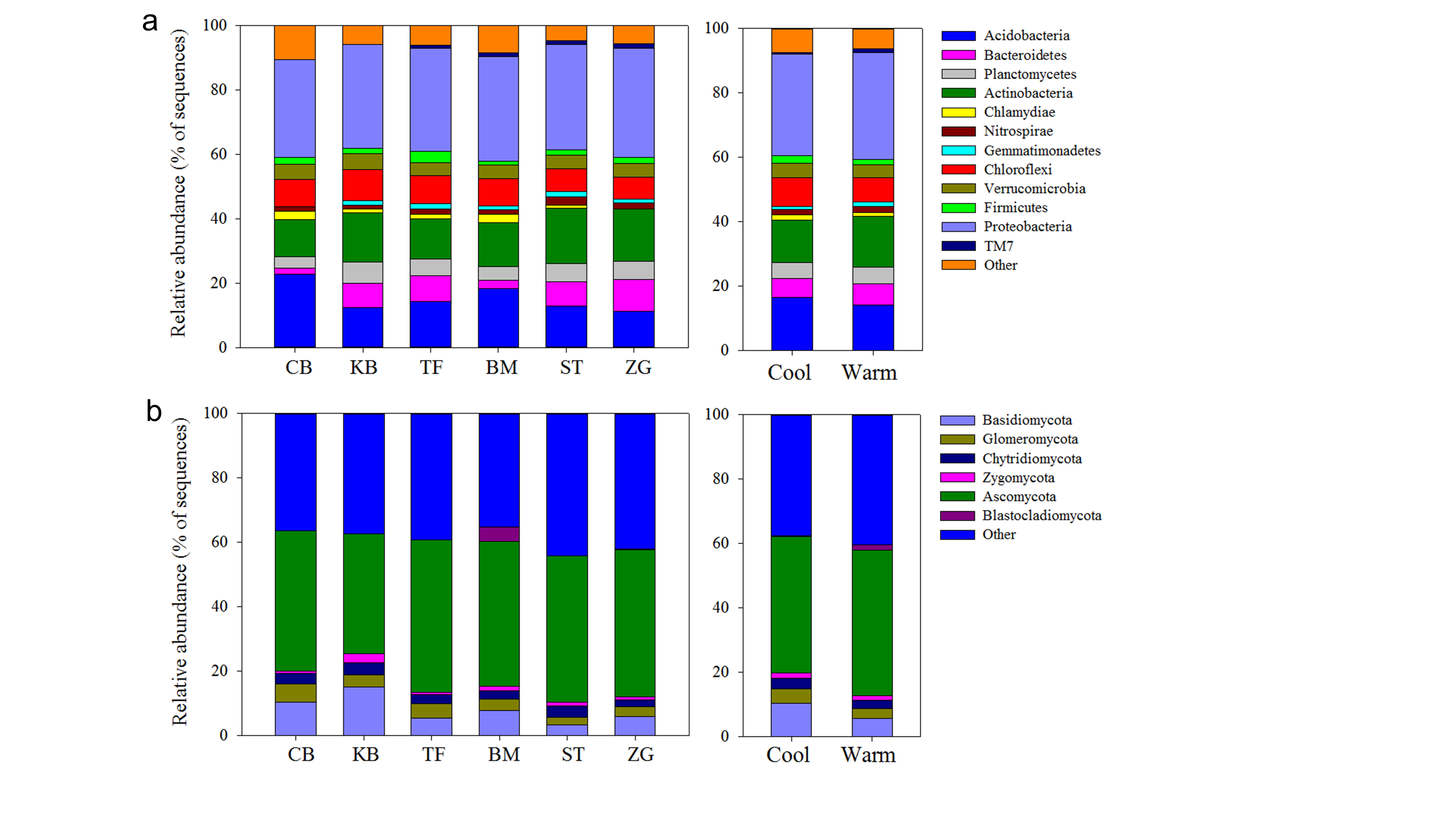

Supplement: S1 Fig — (DOCX) [file pone.0218967.s001.docx]
