# Supplementary material for: Defoliation management and grass growth habits modulated the soil microbial community of turfgrass systems
Source: PLoS One. 2019 Jun 24;14(6):e0218967. doi: 10.1371/journal.pone.0218967 (PMC6590823; doi:10.1371/journal.pone.0218967)

**S2 Fig. LEfSe of cool- and warm-season turfgrass systems.**

Significant differences in the relative abundance of soil bacterial (a) and fungal (b) taxa. Dots from center outward represent phylum, class, order, family, and genus, respectively. The colors of dots indicate more abundant taxa in the respective system. The sizes of dots are proportional to relative abundances of taxa. Only taxa with > 0.5% and 0.025% relative abundance for bacterial and fungal communities, respectively, were included for the analysis.


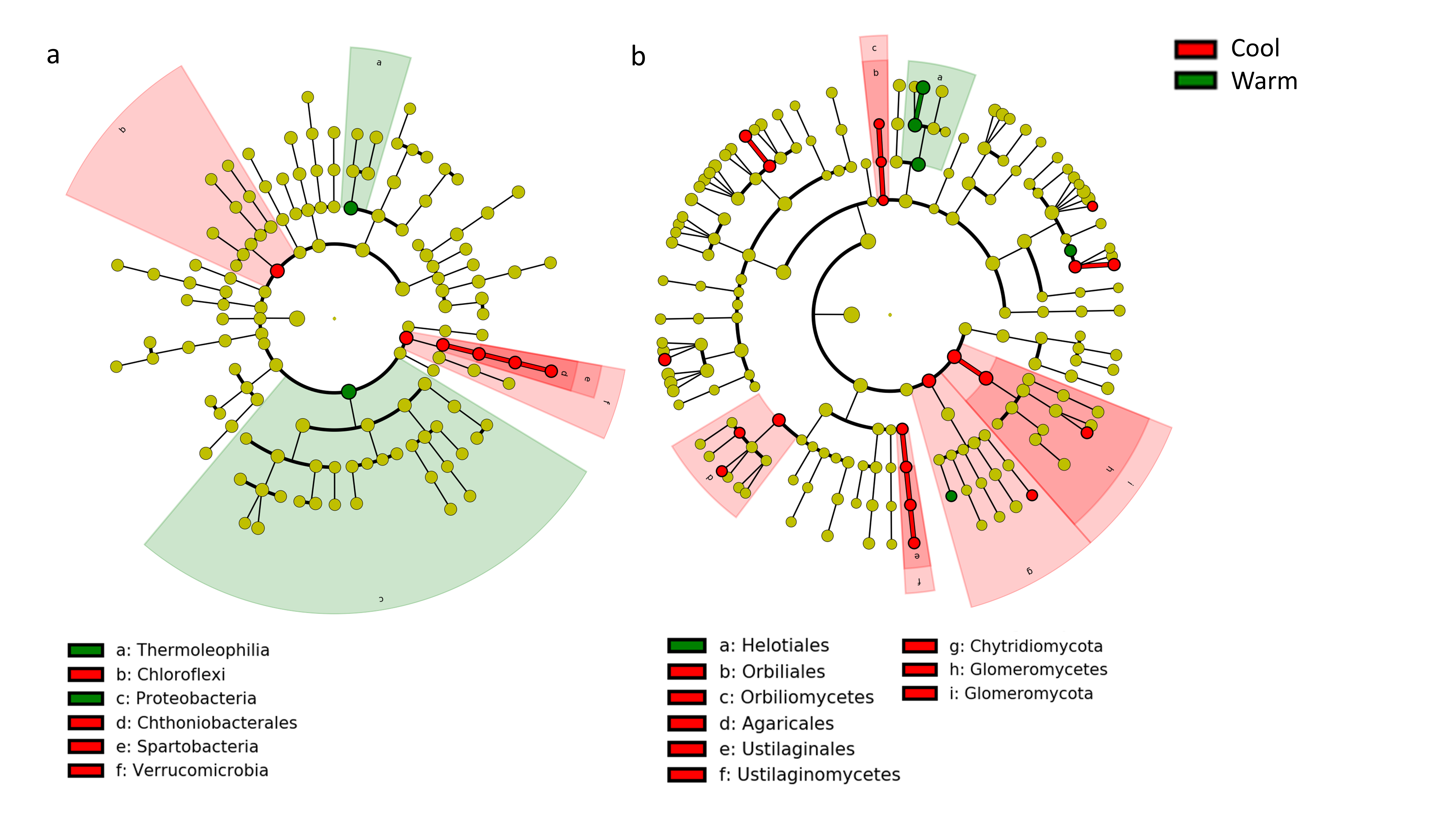

Supplement: S2 Fig — (DOCX) [file pone.0218967.s002.docx]
