# Supplementary material for: Defoliation management and grass growth habits modulated the soil microbial community of turfgrass systems
Source: PLoS One. 2019 Jun 24;14(6):e0218967. doi: 10.1371/journal.pone.0218967 (PMC6590823; doi:10.1371/journal.pone.0218967)

**S3 Fig. Relative abundances of soil bacterial phyla having *nosZ* and *hao* genes.**

Soil bacterial phyla having *nosZ* (a) and *hao* (b) genes in six turfgrass systems (See Table 1 for abbreviation).


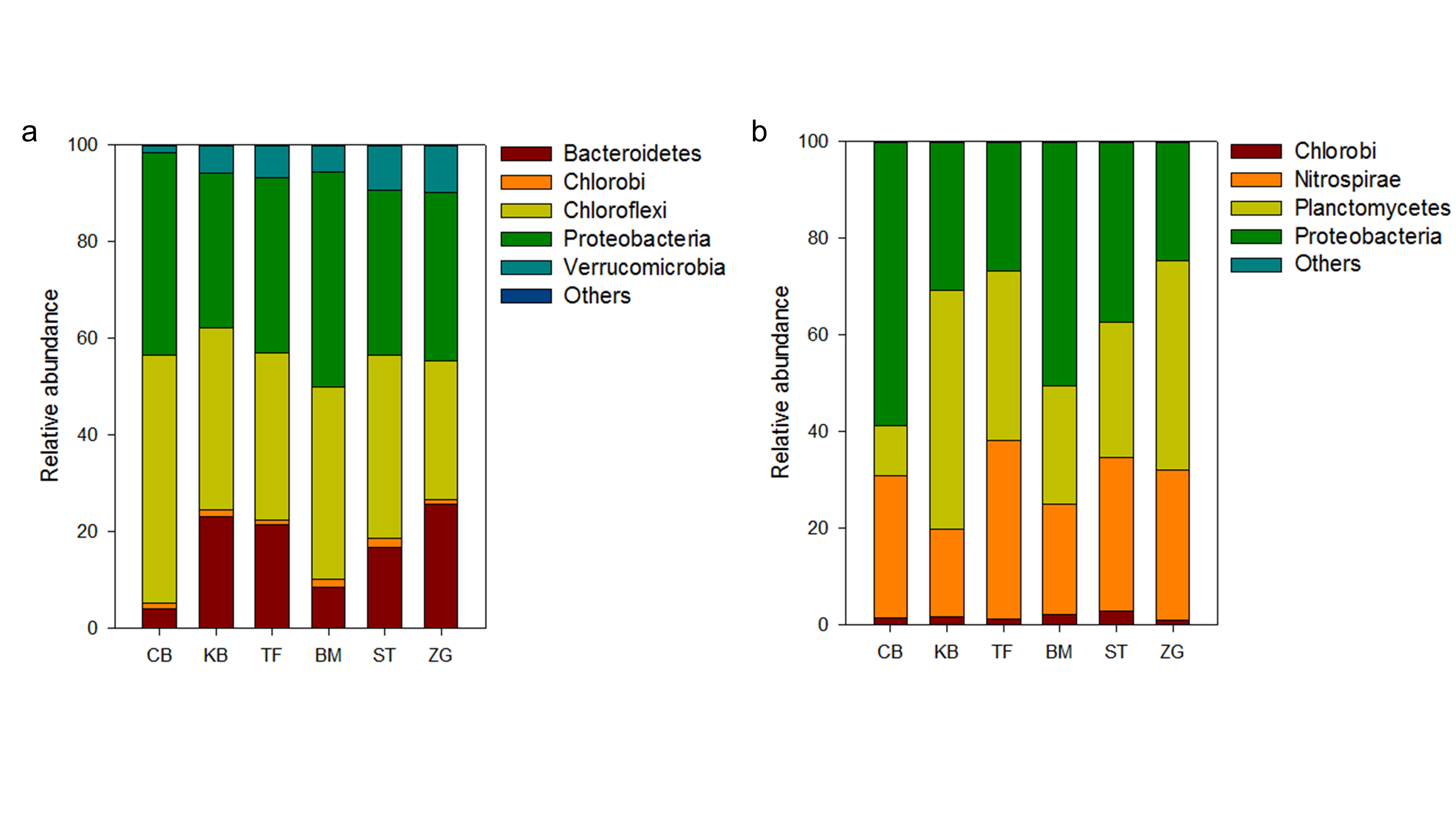

Supplement: S3 Fig — (DOCX) [file pone.0218967.s003.docx]
